# Immune induction strategies to enhance responses to PD-1 blockade: lessons from the TONIC trial

**DOI:** 10.1186/s40425-019-0783-x

**Published:** 2019-11-21

**Authors:** Sandra Demaria, Emanuela Romano, Muriel Brackstone, Silvia C. Formenti

**Affiliations:** 1 Department of Radiation Oncology, Weill Cornell Medicine, 1300 York Ave, Box 169, New York, NY 10065 USA; 2 Department of Pathology and Laboratory Medicine, Weill Cornell Medicine, 1300 York Ave, Box 169, New York, NY 10065 USA; 3 Center for Cancer Immunotherapy, Department of Oncology, INSERM U932, Institut Curie, 26 rue d’Ulm, 75005 Paris, France; 4 Division of Surgical Oncology, London Regional Cancer Program, 790 Commissioners Road East, London, Ontario N6A 4L6 Canada

**Keywords:** Immunomodulation, Immunogenic cell death, tumor burden, Tumor-infiltrating T cells

## Abstract

Programmed cell death protein 1 (PD-1) blockade is only effective in a minority of patients, prompting the search for combinatorial therapies that increase responses. Identifying effective combinations requires lengthy testing and so far has shown few successes. To accelerate progress Voorwerk and colleagues (Nat Med. 25(6):920-8, 2019) used an adaptive trial design to compare 4 short-course therapies (radiotherapy, cyclophosphamide, cisplatin and doxorubicin) for their ability to improve the tumor immune microenvironment and enhance responses to subsequent PD-1 blockade in women with metastatic triple negative breast cancer, a disease with low response rate to PD-1 blockade. They reported the first phase of the trial that enrolled 12 to 17 patients per arm to “pick the winner” induction treatment. Higher objective response rates (ORR) compared to no induction were observed only in the arm containing doxorubicin, which proceeded to phase II. These results raise a number of questions about testing local versus systemic induction treatments and whether sequencing with PD-1 blockade is appropriate in light of evidence supporting concomitant treatment, at least for radiotherapy. Small imbalances in baseline characteristics can also influence results obtained with limited numbers of patients per arm. We hope that these considerations will help future adaptive, signal-finding combination immunotherapy studies.

## Text

In patients with advanced cancer responses to immune checkpoint blockade therapy (ICB) have lasting benefits that can result in improved survival. This has also been shown to be true for breast cancer patients, although overall response rate to single agent PD-1/PD-L1 blockade is quite low [1]. Triple negative breast cancer (TNBC) is an aggressive subtype of breast cancer with on average a higher mutational burden than other subtypes and more frequent infiltration by lymphocytes, features that are both associated with more immunogenic tumors [1]. The response rate of TNBC to PD-1/PD-L1 blockade varies widely in different studies but it is higher if the tumor expresses PD-L1 in the immune infiltrate and ICB is used as first line therapy, reaching 21.4% in the KEYNOTE-086 study [1, 2].

Because of the association of response to PD-1 blockade with the presence of a pre-existing immune-active tumor microenvironment (TME), multiple efforts are ongoing to identify treatments that mobilize and activate anti-tumor T cells and/or shift immune suppression towards immune activation [3]. Combinatorial strategies include standard therapies such as some types of chemotherapy for which there is preclinical evidence showing the induction of immunogenic cell death (ICD) and/or the preferential depletion of regulatory and suppressive immune cell subsets [4]. Based on this rationale, Voorwerk and colleagues [5] selected cyclophosphamide, cisplatin and doxorubicin for testing in the TONIC trial. Unlike other studies that have added PD-1 blockade to standard-of-care chemotherapy, they chose a more original approach: a two-week conditioning treatment, followed by anti-PD-1 therapy and assessment of response. They hypothesized that such a short treatment course would reduce the negative effects of the chemotherapy on T cells while causing enough ICD and pro-immunogenic changes in the TME to “jump-start” anti-tumor immune responses, to increase the response rate to PD-1 blockade in patients with metastatic TNBC. The trial was designed with tumor sampling at baseline, at the end of the induction treatment and after three cycles of PD-1 blockade, allowing for the evaluation of the immunological effects of each intervention on the TME. The fourth induction treatment, focal radiation therapy, was delivered to a single metastasis and tested the effect of local therapy rather than systemic treatment on TME. Similarly to chemotherapy, there is substantial preclinical and some clinical evidence that focal radiation therapy promotes anti-tumor immune responses that can enhance systemic responses to ICB therapy [6, 7]. In the case of radiation, the effects on the TME were evaluated in non-irradiated lesions.

The trial accrued 70 patients randomized between five arms (4 induction treatments and one without induction), 66 completed treatment and were evaluable. The overall objective response rate (ORR) to PD-1 blockade of 20% was higher in this trial than in prior studies. This may be reflective of a high percentage (86%) of patients with PD-L1+ tumors (> 1% on immune cells), and the selection of fit patients with lower tumor burden, based on serum levels of LDH, as discussed by the investigators. When broken down by induction arm, ORR ranged from 8% for radiation and cyclosphosphamide (1/12), 17% for non-induction (2/12), 23% for cisplatin (3/13) to 35% for doxorubicin (6/17), leading the investigators to choose doxorubicin for the phase II expansion.

As pointed out by the investigators, the trial was non-comparative and, despite the limited patient number, it allowed a quick prioritization of treatments based on the discontinuation of the arms with fewer than 3 out of 10 patients exhibiting at least stable disease (SD) after 12 weeks. Several important questions arise from this study. The first pertains to whether the ORR observed reflects the ability of the tested induction treatments to improve responses to PD-1 blockade. Analysis of the post-induction biopsies did not show significant changes compared to baseline in total T cell infiltration, CD8 T cell infiltration or T cell receptor (TCR) clonality in any of the arms. In contrast, patients who exhibited clinical benefit (CR + PR + SD) had significantly higher stromal tumor-infiltrating lymphocytes (sTILs) and CD8 T cell density in the tumor and significantly lower cancer antigen 15–3 and carcinoembryonic antigen serum levels at baseline compared to the patients with progressive disease. Additional analyses of gene signatures in the tumor at baseline showed more T helper 1, B cells and neutrophils in responders than non-responders. Overall, these data indicate that responses to PD-1 blockade were largely pre-determined by the baseline characteristics of the tumor.

Higher TCR clonality and T cell infiltration were seen in responders than non-responders after three cycles of PD-1 blockade, and when broken down by induction therapy there was a trend toward a greater increase compared to baseline in the doxorubicin and cisplatin arms, recognizing the limited sample size (*n* = 3 for radiation and cisplatin, *n* = 5 for doxorubicin, *n* = 6 for cyclophosphamide and no-induction). The intratumoral TCR repertoire diversity was significantly increased compared to baseline in patients in the doxorubicin arm, but only 1 out of 5 patients with available data had clinical benefit, making it difficult to understand its biological meaning. Thus, it appears that among responders, PD-1 blockade was driving an expansion of the pre-existing T cell responses.

These considerations raise the question of why there was such a difference in ORR between the arms. Part of the answer may lie in the fact that in small patient cohorts any imbalance in baseline characteristics can result in a large effect on clinical outcome. Despite the quality of the trial design, there was a slightly higher proportion of patients (6/17) receiving their first line treatment among those enrolled in the doxorubicin arm compared to the other induction arms. Given the objective improvement in response rate to PD-1 blockade in untreated versus previously treated metastatic patients observed in the KEYNOTE-086 study [2], it is conceivable that this variable might have favored the doxorubicin induction arm. There was also some imbalance in the proportion of tumors with > 5% sTILs at baseline, lowest in the radiation group (36% in radiation versus 53–69% in the other groups). While this factor might have reduced the likelihood of responses to PD-1 in this group, by itself cannot explain the results, since the highest sTILs percentage was observed in the cyclophosphamide group.

Three of the induction treatments were systemic and two patients, one in the cisplatin and one in the doxorubicin group, had PR at the end of the induction treatment, suggesting that some tumors were particularly sensitive to the chemotherapy itself. Despite little evidence of changes in the immune infiltrate in any of the arms after induction, immune related gene signatures showed an enrichment using a Bayesian model after doxorubicin and cisplatin treatment that passed multiple testing correction in the doxorubicin group. This was not observed in the radiation group. A major difference between radiation and the other induction treatments is that a single metastatic lesion was treated with radiation but the effect of that radiation on the tumor immune microenvironment was measured in non-irradiated lesions. Since radiation alone is expected to modulate anti-tumor responses locally rather than systemically, it was predictable that the post-induction biopsies would not differ from the biopsies taken from the tumors in the no induction arm. Combination of radiation and ICB treatment is essential for systemic anti-tumor effect. Indeed, preclinical evidence shows that the synergy of radiation with PD-1 blockade is lost when the latter is started a week after completing radiation treatment [8]. Therefore, the sequential design of the treatment failed to consider that, at least for radiation, concomitant administration is likely to be essential to optimally leverage its synergy with ICB.

Induction with radiation was done using a hypo-fractionated radiation regimen (a total dose of 24 Gy delivered by 8 Gy fractions) that was shown to induce systemic anti-tumor responses when combined with concomitant administration of anti-CTLA-4 or anti-PD-1 in pre-clinical models, and anti-CTLA-4 in lung cancer patients [6, 7]. Mechanistic studies have demonstrated that the efficacy of hypo-fractionated radiation is related to its ability to activate the type I interferon pathway in the tumor [6]. Clinical data show that an increase in serum interferon-β is detectable after radiation and correlates with objective systemic response to radiation and CTLA-4 blockade in lung cancer patients [7]. Therefore, it would be interesting to know if such response was induced in the TNBC patients that received radiation in the TONIC trial. In vitro, 8 Gy radiation doses given on three consecutive days induced the secretion of interferon-β by human TNBC cells. In vivo, irradiation of tumors obtained by implantation of the same TNBC cells upregulated the expression of type I interferon-stimulated genes [6]. However, expression of the cytosolic DNA sensor cGAS, and its adaptor STING, both of which are required for radiation-induced interferon-β induction, are variable in many tumors including breast cancer [9], and may be a factor influencing the ability of radiation to prime anti-tumor immune responses.

Finally, given the tumor heterogeneity in metastases of advanced breast cancer [10] the expectation that a local treatment of a single metastasis might induce systemically effective anti-tumor immune responses may not be realistic. A more realistic approach for this disease setting may require the irradiation of multiple tumor sites. Results of the phase II TONIC trial will show if doxorubicin proves as an effective induction agent. For radiation the jury is still out, awaiting testing its role as an “inducer” of in situ vaccination in trials that will take into consideration its optimal use.

Multiple other strategies have been used to enhance responses to PD-1/PD-L1 blockade in pre-clinical and early clinical studies, including several intra-tumoral immune modulators and targeted agents [11]. The adaptive, signal-finding combination immunotherapy design used in the TONIC trial could provide an efficient model to identify active combinations, especially when coupled with correlative studies to investigate mechanisms of action. However, we believe that a careful patient selection that takes into consideration the tumor burden, presence of TILs, and prior lines of therapy, as proposed by Wein and colleagues [1] is crucial to improve the interpretation of the results of this type of studies.

## Data Availability

Not applicable.

## Abbreviations

CR: Complete Response
ICB: Immune Checkpoint Blockade Therapy
ORR: Objective Response Rates
PD-1: Programmed Cell Death Protein 1
PD-L1: Programmed Cell Death Protein Ligand 1
PR: Partial Response
SD: Stable Disease
sTILs: Stromal Tumor-Infiltrating Lymphocytes
TCR: T Cell Receptor
TME: Tumor Microenvironment
TNBC: Triple Negative Breast Cancer
